# Leucine-rich diet supplementation modulates foetal muscle protein metabolism impaired by Walker-256 tumour

**DOI:** 10.1186/1477-7827-12-2

**Published:** 2014-01-03

**Authors:** Bread Cruz, Maria CC Gomes-Marcondes

**Affiliations:** 1 Laboratory of Nutrition and Cancer, Department of Structural and Functional Biology, Institute of Biology, State University of Campinas - UNICAMP Campinas, 13083862 Sao Paulo, Brazil

**Keywords:** Foetal protein metabolism, Nutritional supplementation, Leucine, Walker tumour, Proteasome

## Abstract

**Background:**

Cancer-cachexia induces a variety of metabolic disorders of protein turnover and is more pronounced when associated with pregnancy. Tumour-bearing pregnant rats have impaired protein balance, which decreases protein synthesis and increases muscle breakdown. Because branched-chain amino acids, especially leucine, stimulate protein synthesis, we investigated the effect of a leucine-rich diet on protein metabolism in the foetal gastrocnemius muscles of tumour-bearing pregnant rats.

**Methods:**

Foetuses of pregnant rats with or without Walker 256 tumours were divided into six groups. During the 20 days of the experiment, the pregnant groups were fed with either a control diet (C, control rats; W, tumour-bearing rats; Cp, rats pair-fed the same normoprotein-diet as the W group) or with a leucine-rich diet (L, leucine rats; LW, leucine tumour-bearing rats; and Lp, rats pair-fed the same leucine-rich diet as the LW group). After the mothers were sacrificed, the foetal gastrocnemius muscle samples were resected, and the protein synthesis and degradation and tissue chymotrypsin-like, cathepsin and calpain enzyme activities were assayed. The muscle oxidative enzymes (catalase, glutathione-S-transferase and superoxide dismutase), alkaline phosphatase enzyme activities and lipid peroxidation (malondialdehyde) were also measured.

**Results:**

Tumour growth led to a reduction in foetal weight associated with decreased serum protein, albumin and glucose levels and low haematocrit in the foetuses of the W group, whereas in the LW foetuses, these changes were less pronounced. Muscle protein synthesis (measured by L-[3H]-phenylalanine incorporation) was reduced in the W foetuses but was restored in the LW group. Protein breakdown (as assessed by tyrosine release) was enhanced in the L and W groups, but chymotrypsin-like activity increased only in group W and tended toward an increase in the LW foetuses. The activity of cathepsin H was significantly higher in the W group foetuses, but the proteolytic calcium-dependent pathway showed similar enzyme activity. In parallel, an intense oxidative stress process was observed only in the group W foetuses.

**Conclusions:**

These data suggested that the proteasomal and lysosomal proteolytic pathways and oxidative stress are likely to participate in the process of foetal muscle catabolism of Walker’s tumour-bearing pregnant rats. The present work shows that foetal muscle can be protected by supplementation with a leucine-rich diet.

## Background

Pregnancy concomitant with cancer occurs in approximately one in one thousand women, but this combination is responsible for one-third of all maternal deaths [1,2]. Relatively few relevant reports exist in the scientific literature regarding the consequences of tumour growth for the maternal host and the foetal body. Our previous studies showed that tumour-bearing dam rats displayed approximately 70% reabsorption and 50% foetal death [3-6]; in this work, we focused on foetal growth because there is little data about the effects on foetal homeostasis and damage when the mother suffers from a tumour [7].

Tumour growth promotes extensive biochemical and metabolic changes in the host organism, leading to the process of cachexia and culminating in deep catabolism of lean mass [8-10]. As a result, the host demonstrates marked weight loss, which is not minimised by forced feeding. To study these all these processes experimentally, Walker-256 tumour is a good experimental model of cachexia [9,10]. In addition, pregnancy causes physiological changes that ensure foetal growth and development until the term pregnancy and ensures an adequate energy supply to the foetus and after birth [11]. Additionally, during pregnancy, increased oxidative stress can cause pre-eclampsia and intrauterine foetal growth retardation [12], which may be promoted by tumour development [13].

Branched-chain amino acids (BCAAs) are essential for humans by promoting cell function and regulating protein metabolism [14]. BCAAs, especially leucine (Leu), have been studied because of their cell-signalling properties; they can stimulate protein synthesis [14] and decrease protein catabolism [6,14,15]. The oral administration of exogenous leucine stimulates protein synthesis in isolated or perfused rat muscles in vivo [16]. Moreover, clinical trials and studies in animals have shown that supplementation with BCAAs increases the balance of nitrogen and improves health after tissue damage, such as that due to burns, radiation, post-surgical stress, sepsis and cancer [17]. Studies have shown that the use of complementary therapies, as nutritional supplementation, improves the clinical status and especially the quality of life of cancer patients [18-21]. Meanwhile, maternal metabolism is directed to ensure the nutritional supply for the foetus and adjust foetal growth, but the combination of the metabolic and biochemical changes during pregnancy and cancer jeopardises the foetus and mother. Based on this, and more importantly because there are few studies about the foetal consequences of cancer development, this study investigated the effects of Walker-tumour growth on protein catabolism in foetal muscle, especially on proteolytic enzymes, anti-oxidant enzymes and lipid peroxidation and examined whether nutritional supplementation with leucine modulated the damage effects on foetal growth.

## Methods

### Animals

We studied foetuses (20 days of gestational age) that were obtained from pregnant adult Wistar rats. Female Wistar rats (90 days old) were obtained from the Animal Facilities Centre/UNICAMP and received food and water ad libitum under controlled light–dark cycles (12/12 hours each) at a constant temperature (22 + 2°C). The female rats were bred with adult males (4:1 proportion) in collective cages for 12 hours [22]. After the first day of pregnancy was determined by vaginal smears, the pregnant rats were distributed into groups that either received or did not receive a tumour implant and were either administered nutritional supplementation or administered normal diet. Walker carcinoma 256 tumour cells (2.5 × 10^5^ viable cells counted using the trypan blue method) were injected subcutaneously on the 2^nd^ day of pregnancy into the right flank of the experimental rats. The pregnant rats in the control groups received an equivalent volume of saline solution (0.5 mL) in the subcutaneous tissue of their right flanks. The handling of pregnant females with tumours was previously described in protocol #657-1, which was approved by the Ethics in Animal Experiments (CEUA-IB-UNICAMP).

### Diet

The semi-purified, isocaloric diets were defined as either normal (C - containing 18% protein [23]) or leucine-rich (L - containing 18% protein plus 3% L-leucine, which represents a high percentage of this amino acid). Both diets contained 70% carbohydrates (sucrose, starch and dextrin), 7% fat (soybean oil) and 5% fibre (micro-cellulose purified). The diets were supplemented with a mixture of vitamins, minerals, cysteine and choline. The control diet contained 1.6% L-leucine, and the leucine-rich diet contained 4.6% L-leucine; these diets were identical to those used in our previous experimental studies [5,24-26].

### Experimental protocol

The pregnant rats were distributed into 6 experimental groups according to tumour implant status and nutritional supplementation. Pair-fed groups were created to evaluate the effects of food intake reduction observed in tumour-bearing groups [27,28]. At least three foetuses per mother were collected to generate a pooled sample (3 foetuses/dam) for each group. The minimum number of mothers per group was at least eight animals.

### Groups

• Rat foetuses (C) from pregnant rats subjected to a normal diet.

• Rat foetuses (L) from pregnant rats subjected to a leucine-rich diet.

• Rat foetuses (W) from Walker tumour-bearing pregnant rats subjected to a normal diet.

• Rat foetuses (LW) from Walker tumour-bearing pregnant rats subjected to a leucine-rich diet.

• Foetuses from pair-fed pregnant rats (Cp) subjected to the normal diet and paired with group W.

• Foetuses from pair-fed pregnant rats (Lp) subjected to a leucine-rich diet and paired with group LW.

The pregnant rats were maintained in collective cages during the experimental period, except for the pair-fed rats, which remained in metabolic cages and received food intake in accordance with the tumour-bearing groups, as previously determined in our lab studies [4]. After 20 days of pregnancy, the rats were sacrificed by cervical dislocation, and the foetuses and placentas were removed, dissected and weighed. The gastrocnemius foetal muscles were dissected and weighed to assess protein synthesis and degradation, and the activities of oxidative stress enzymes and malondialdehyde were assayed in foetal muscles. The foetal muscle homogenates were also analysed for the following enzyme activities related to the protein degradation process: the chymotrypsin-like enzyme of the ubiquitin-proteasome pathway, the cathepsin enzyme of the lysosomal pathway and the calpain enzyme of the calcium-dependent pathway. We also evaluated the expression of the 20S subunit of the ubiquitin-proteasome system.

### Protein synthesis and degradation analyses

The synthesis and degradation of proteins was assessed in pooled, foetal gastrocnemius muscles from each mother. For statistical analysis, the number of pregnant rats was at least 8 for the control groups and 10 to 12 for the tumour-bearing groups.

Protein synthesis assays were performed using methods that were previously adapted in our laboratory [6,28,29]. Pooled gastrocnemius muscles from both posterior paws of the foetuses (at least 3 foetuses per mother and were used at least 8 mothers per group) were dissected, placed in tubes for perfusion with KHB buffer (Krebs-Henseleit bicarbonate, 110 mM NaCl, 25 mM NaHCO_3_, 3.4 mM KCl, 1 mM CaCl_2_, 1 mM MgSO_4_ and 1 mM KH_2_PO_4_; pH 7.4) supplemented with 5.5 mM glucose and 0.01% (w/v) albumin and incubated at 37°C under continuous agitation in a 95% O_2_ and 5% CO_2_ environment for 30 minutes. After this period, fresh KHB buffer supplemented with 5 μCi of L[^3^H]-phenylalanine (Amersham, UK) and 60 mM cold phenylalanine was added. After 2 hours of incubation, the muscles were removed from the medium, dried, weighed, frozen in liquid nitrogen and stored at -80°C. The muscles were homogenised in 30% trichloroacetic acid (TCA) and centrifuged at 11,000 × *g* for 15 min at 4°C. The resulting pellet was washed twice with TCA (10%) to eliminate the radioactivity of the supernatant. The pellet was treated with 1 N NaOH and incubated at 40°C for 30 minutes. The protein content of the aliquots was then analysed [30], and the total β emission radioactivity was assessed by liquid scintillation using beta-counting equipment (Beckman LS 6000 TA, Fullerton, CA, USA). The rates of protein synthesis, expressed as nanomoles of phenylalanine incorporated per hour per milligram of muscle protein, were calculated by dividing the amount of radioactivity incorporated into the foetal muscle protein over a 1-h period by the specific radioactivity of the phenylalanine in the incubation medium.

A protein degradation assay was also adapted by our laboratory [31] by measuring the amount of tyrosine that was released into the muscle incubation solution. Each pooled foetal gastrocnemius muscle sample (3 foetuses per mother/group) was incubated for 30 min at 37°C in RPMI 1640 medium. After this period, the muscles were incubated in KHB solution containing cycloheximide (a protein synthesis inhibitor) for 2 hours at 37°C in a 5% CO_2_ and 95% O_2_ environment. After incubation, the medium aliquots were treated with 30% TCA and centrifuged at 4,500 × *g* for 10 min to assess the tyrosine content using a fluorometric assay utilising 20% 1-nitroso-2-naphthol reagent and nitric acid [32].

### Biochemical assays to assess the muscle proteolytic pathways and oxidative stress

The foetal gastrocnemius muscle samples were homogenised in homogenising buffer (20 mM Tris, 1 mM DTT, 2 mM ATP and 5 mM MgCl_2_), resolved on 12% acrylamide-SDS gels and transferred to nitrocellulose membranes. A primary monoclonal antibody against the 20S subunit of the proteasome (Affinity, England) (1:1,000 dilution) was used, and a secondary goat anti-mouse antibody was used to react with the primary antibody (Santa Cruz, USA) (1:1,000). The chemiluminescence reaction was performed using ECL reagents (Amersham), and the quantification of protein-band density was carried out using Image Capture (Amersham) and Gel Pro II-Plus software (Silver Spring, MD, USA).

The foetal muscle homogenates was also analysed for total protein content [30] and for chymotrypsin-like, cathepsin and calpain enzyme activity. Chymotrypsin-like activity, which is the proteolytic activity of the ubiquitin-proteasome pathway, was evaluated using the substrate N-Succinyl-Leu-Leu-Val-Tyr-7-Amino-4-Methylcoumarin diluted in dimethyl sulfoxide (DMSO) and Tris buffer (pH 8.0) and assessed using the fluorometric method at 360 nm excitation and 460 nm emission [33]. The activity of cathepsin, which is a lysosomal proteinase, was evaluated using a fluorometric method and the substrate benzyloxycarbonyl-phenylalanine- arginine 4-methyl-7-coumarilamide at 340 nm excitation and 460 nm emission [34]. Calpain, which is a proteinase involved in calcium-dependent protein degradation, was measured by incubating the samples in reaction buffer with casein for 5 minutes followed by the addition of 5 mM CaCl_2_ and subsequently reading the absorbance at 500 nm in a spectrophotometer according to the previously described colourimetric methodology [34].

Aliquots of the muscle-homogenate supernatant were analysed for glutathione-S-transferase (GST) activity based on the conjugation of 1-chloro-2,4-dinitrobenzene (CDNB; Sigma) with glutathione, and the activity was expressed as nmol·μg protein^-1^·min^-1^ using an extinction coefficient of 9.6 as previously described [35]. The catalase activity was measured as described by Cohen [36], and the results are expressed as nmol/min/mg protein. Alkaline phosphatase activity was measured using 37 mM 4-nitrophenyl disodium phosphate (p- NPP) and expressed in nmol/min/mg protein [37]. The amount of the lipid peroxidation product, malondialdehyde (MDA), was determined by incubating the samples with MPO (n-methyl-2-phenylindole; Sigma) and measuring the absorbance at 590 nm; the results are expressed as nmol·μg protein^-1^[38].

After decapitation, foetal blood was collected using a microcapillary tube and then centrifuged to assay the haematocrit and obtain the foetal serum. The total protein concentration in the foetal serum was determined using the Biuret test, which is a colourimetric reaction that was read at 540 nm [39]. The albumin concentration in the foetal serum was determined using colourimetric methodology by reading a bromocresol green reaction at 630 nm [40]. The glucose level was quantified using an enzymatic reaction based on glucose-oxidase [41].

### Statistical analysis

The results are reported as the means and standard error. The data were analysed using two-way ANOVA tests to detect the effects of diet, tumours, and the pair-feeding scheme on the foetal parameters (weight, enzyme activities and protein synthesis and degradation). The comparisons within the control and tumour-bearing groups were performed using one-way ANOVA followed by Bonferroni multiple-comparison tests [42]. The calculations were performed using GraphPad Prism 3.0 software (GraphPad Inc., San Diego, CA, USA), and values of P < 0.05 indicate statistical significance.

## Results

**Figure 1:**
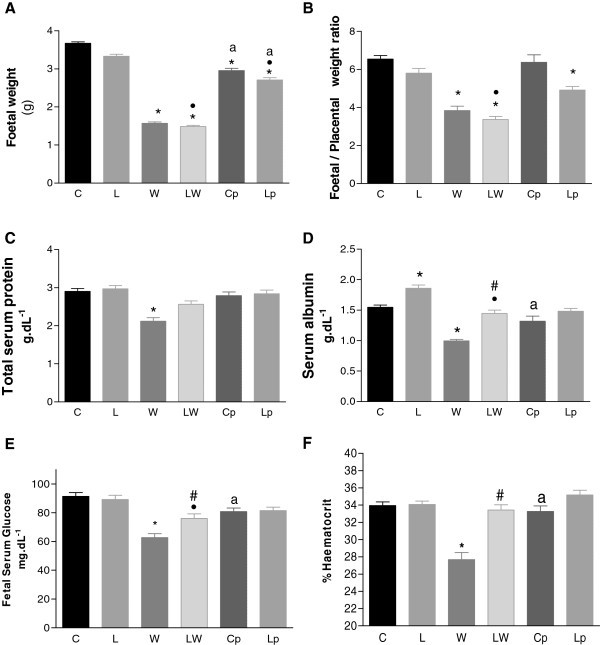
**Foetal tissues.** Foetal weight **(A)**, the foetal/placental weight ratio **(B)**, total serum protein **(C)**, albumin **(D)** and glucose **(E)** levels and haematocrit **(F)** of foetuses from Walker 256 carcinoma-bearing pregnant rats that were administered a leucine-rich or control diet. Legend: C – control rats; L – rats fed a leucine-rich diet; W – tumour-bearing rats; LW – tumour-bearing rats fed a leucine-rich diet; Cp – rats subjected to a similar, paired-nutrition intake of rats in group W; and Lp – rats subjected to a leucine-rich diet and nutrition that was paired to the intake of rats in Group LW. * P < 0.05, significantly different compared to group C. • P < 0.05, significantly different compared to group L. ^a^ P < 0.05, significantly different compared to groups W and LW. # P < 0.05, significantly different from group W.

In the present study, the foetuses of tumour-bearing pregnant rats suffered similar effects due to tumour growth as those observed in tumour-bearing animals [5,6,15,25,27]. The weight of the foetuses in the W and LW groups was reduced (Figure 1A), and in this case the tumour effects account for 83% of the total variation, indicating that this low foetal weight is extremely significant. Consistent with this reduced foetal weight, the ratio of the foetal weight to the placental weight, which is indicative of the efficiency of placental transport, was decreased in both tumour-bearing groups and in the leucine pair-fed foetuses, especially when compared to the control rats (Figure 1B). These data show that the tumours and the pair-feeding scheme accounted for this impaired effect (P < 0.0002). The reduction in foetal weight occurred in parallel with the group with the lowest concentration of total foetal serum protein, group W, which was 27% lower than that of the control group, C (Figure 1C). In contrast, the total foetal serum protein of the LW group was similar to that of the control group (C) and higher than that of the W group (P = 0.0029). The serum albumin levels of the rats in the tumour-bearing group (W) were reduced by approximately 31% compared to those in group LW (P < 0.0001), which had a similar value to that of the control group (Figure 1D). Although the albumin-serum content was 20% higher in group L (under nutritional supplementation) than in group C, the leucine-rich diet did not prevent the reduction in albumin levels in the foetuses from tumour-bearing rats, as observed when comparing the LW and L groups (Figure 1D, P < 0.0001). Similarly, we observed a reduction in glucose levels in tumour-bearing foetuses, and those in the W groups showed a severe decrease in glucose levels (Figure 1E). Furthermore, the haematocrit was reduced only in the W group (Figure 1F). These data show that diet accounted for 46.7% of the total variance (P < 0.0001), modulating the effect of Walker tumour development.

**Figure 2:**
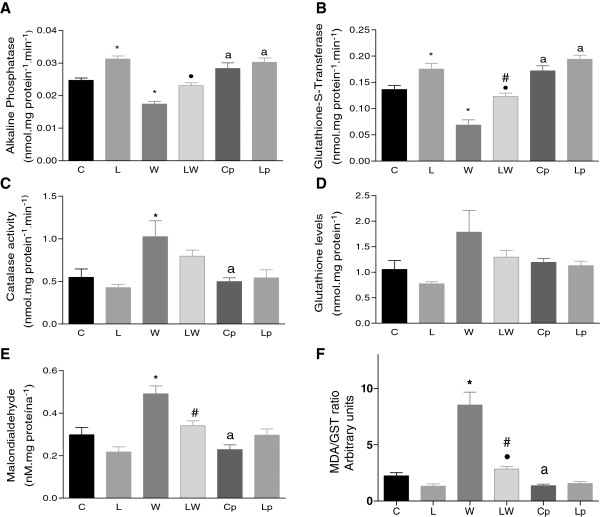
**Foetal muscles.** Alkaline phosphatase **(A)**, glutathione-S-transferase **(B)**, catalase **(C)**, glutathione **(D)**, malondialdehyde **(E)** levels and the MDA/GST ratio **(F)** of the foetal muscle from Walker 256 carcinoma-bearing pregnant rats that were subjected to a leucine-rich or control diet. Legend: C – control rats; L – rats fed a leucine-rich diet; W – tumour-bearing rats; LW – tumour-bearing rats fed a leucine-rich diet; Cp – rats subjected to a similar, paired-nutrition intake of rats in group W; and Lp – rats subjected to leucine-rich diet and nutrition that was paired to the intake of rats in Group LW. * P < 0.05, significantly different compared to group C. • P < 0.05, significantly different compared to group L. ^a^P < 0.05; significantly different compared to groups W and LW. # P < 0.05, significantly different from group W.

Tumour growth affected anti-oxidative enzyme activities and increased lipid peroxidation (malondialdehyde) in foetal gastrocnemius muscles (Figure 2). Alkaline phosphatase activity could be used to test muscle-cell function [37], which was decreased in the W foetuses, but leucine supplementation led to a similar level in the LW group. In contrast, the L, Cp and Lp groups displayed increases in alkaline phosphatase activity compared to the control or tumour-bearing groups (Figure 2A). In comparison to the C group, glutathione-S-transferase activity was reduced in the W group but was similar in the LW group and increased in the L, Cp and Lp groups (Figure 2B). Catalase activity increased significantly only in the W group (Figure 2C), and glutathione levels tended to increase only in the W group (Figure 2D). The product of oxidative stress, as expressed by the malondialdehyde level, increased only in the W group (Figure 2E), and the MDA/GST ratio was increased 3-fold in the W group compared to the LW group (Figure 2F). These data show that diet accounted for 27% of the total variance in enzyme activity, modulating the tumour changes to foetal antioxidant enzyme activities.

**Figure 3:**
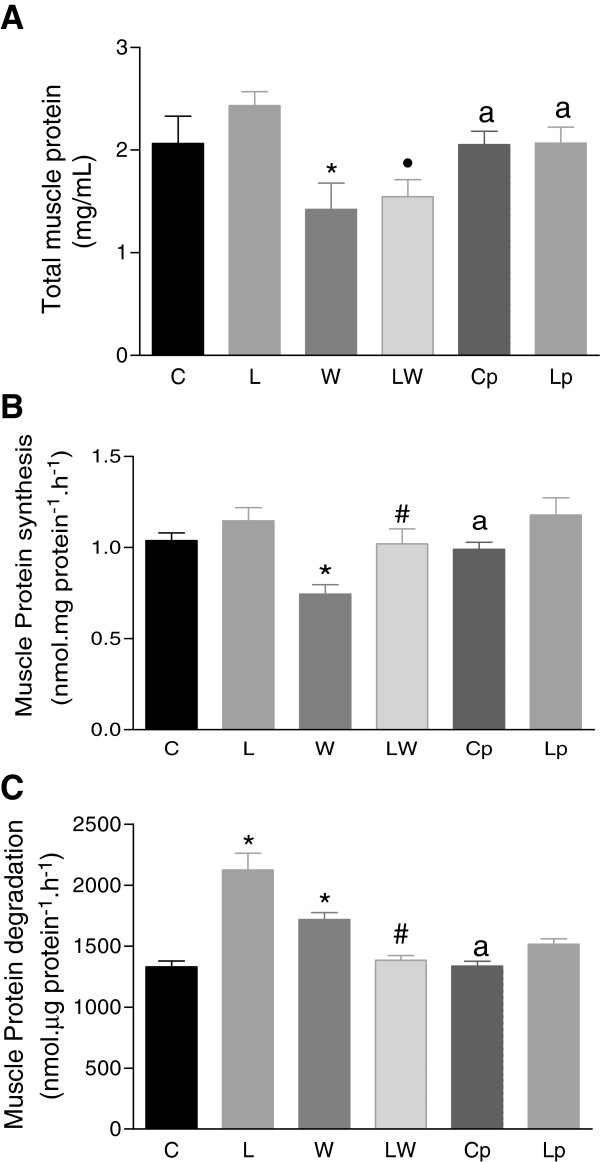
**Foetal muscle protein synthesis and degradation.** Total foetal muscle protein (μg · mg^-1^) **(A)**, muscle protein synthesis (nmol · μg protein^-1^ · h^-1^) **(B)** and muscle protein degradation (nmol · μg protein^-1^ · h^-1^) **(C)** in foetuses of tumour-bearing pregnant rats that were subjected to a leucine-rich or control diet. Protein synthesis was assessed by measuring phenylalanine incorporation (for details, see Methods), and protein degradation was assessed by the amount of tyrosine that was released from perfused muscle (for details, see Methods). Legend: C – control rats; L – rats fed a leucine-rich diet; W – tumour-bearing rats; LW – tumour-bearing rats fed a leucine-rich diet; Cp – rats subjected to a similar, paired-nutrition intake of rats in Group W; and Lp – rats subjected to a leucine-rich diet and nutrition that was paired to the intake of the rats in Group LW. * P < 0.05; significantly different compared to group C. • P < 0.05; significantly different compared to group L. ^a^P < 0.05; significantly different compared to groups W or LW. # P < 0.05, significantly different from group W.

The total muscle protein content (Figure 3A) was decreased in both of the Walker tumour-bearing groups (W and LW) compared to the control groups (C and L). Despite the food restriction that was imposed by pair feeding, no differences in the foetal muscle protein content in the pair-fed groups (Cp and Lp) were observed. Foetal muscle protein synthesis, as determined by the incorporation of phenylalanine (L-[^3^H]-Phe), was approximately 27% lower in the foetuses of tumour-bearing pregnant rats (W) than in the LW group (P = 0.0103) and other groups (Figure 3B). As a result of leucine supplementation, muscle protein synthesis recovered in the LW group, and this change was significantly different from that of the W group; diet significantly modulated the tumour effect (P = 0.0005). In addition, the malnutrition factor that was imposed by food restriction, measured in the pair-fed groups (Cp and Lp), had no effect on the protein synthesis process (Figure 3B).

**Figure 4:**
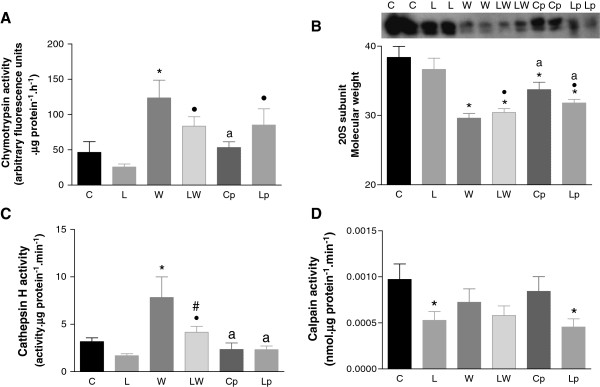
**Foetal muscle enzymes related to protein degradation.** Muscle chymotrypsin enzyme activity **(A)**, the 20S proteasome subunit **(B)**, cathepsin H enzyme activity **(C)**, and calpain activity **(D)** in foetal muscle from tumour-bearing pregnant rats that were subjected to a leucine-rich or control diet. Representative image showing the expression of the 20S proteasome subunit from each different animal per group; the 20S expression was corrected for actin expression, which was used as a loading control (data not shown) (for details, see Methods). The minimum number of animals used per group was 8. Legend: C – control rats; L – rats fed a leucine-rich diet; W – tumour-bearing rats; LW - tumour-bearing rats fed a leucine-rich diet; Cp – rats subjected to a paired-nutrition intake identical to the rats in Group W; and Lp – rats subjected to a leucine-rich diet whose nutrition was paired to the intake of the rats in Group LW. * P < 0.05; significantly different compared to group C. • P < 0.05; significantly different compared to group L. a P < 0.05; significantly different from groups W and LW. # P < 0.05, significantly different from group W.

Foetal muscle protein degradation was evaluated by the amount of released tyrosine and was approximately 31% higher in the W group than in the control group C (Figure 3C); in this case, the diet also accounted for a significant modulation of tumour effect (P = 0.0038). The chymotrypsin-like enzymatic activity, which is the catalytic portion of the ubiquitin-proteasome, was increased approximately 3.9-fold in group W, and despite having tumours, the foetuses from pregnant rats that received a leucine-rich diet (LW) showed a less-pronounced increase in the chymotrypsin-like activity compared to the rats in group W. These differences were near statistical significance (Figure 4A; P = 0.0803). The pair-fed groups (Cp and Lp) showed an increase in chymotrypsin-like enzyme activity that was similar to that observed in the LW group when compared to the L group. Furthermore, the expression of the 20S subunit of the ubiquitin-proteasome was decreased in the tumour-bearing groups (W and LW) and the pair-fed groups (Cp and Lp) (Figure 4B). Cathepsin H, which is one of the enzymes involved in the lysosomal proteolytic pathway, showed significantly increased activity in the W group, but this increase was less pronounced in the LW group (Figure 4C) (P < 0.047); in this case, the diet modulated the effect of tumour growth. The calcium-dependent proteolytic pathway showed no difference in its calpain activity between the tumour-bearing groups when compared to the C group, but the L and Lp groups showed decreased activity in relation to the values observed in group C rats (Figure 4D).

## Discussion

Maternal physiological adaptations are impaired by cancer development and can lead to impaired foetal growth and development. Here, we clearly show that in the presence of Walker-256 tumour growth, foetal development was jeopardised, especially with respect to muscle protein metabolism linked to increased proteolysis and inhibited protein synthesis. These effects were likely associated with the lack of an anti-oxidative response, which reduced foetal weight. More importantly, we observed that nutritional supplementation with leucine led to a marked modulatory effect that minimised foetal proteolysis, maintained the balance of similar levels of protein synthesis and degradation, and restored the anti-oxidant responses.

Previous studies found that the presence of a tumour affected foetal weight [4,5]. This decrease in foetal weight was related to a reduction in placental weight, which may be related to changes in placental tissue, and decreased the foetal/placental weight ratio (Figure 1), leading to impaired foetal development. Foetal growth in rats is more intense during the third phase of pregnancy (19^th^ - 21^st^ day), which coincides with the greatest tumour weight [3,5]. The high activity of tumour cells that promotes exponential growth occurs during the same period of foetal growth. Thus, we verified that, at the time points when the tumour weighed approximately 10 to 12% of the maternal body weight, the W foetuses also displayed reductions in body weight, total serum protein, albumin and glucose that was coupled with a decline in muscle protein synthesis and increased proteolysis. All of these findings are also the main metabolic changes that occur during tumourigenesis [25], as was found in the group W foetuses. Proteolysis is the main process during cancer-cachexia, and this can be assessed by a decrease in albumin content, followed by the catabolism of skeletal muscle cells, promoting atrophy and apoptosis [43,44]. Although the total muscle protein decreased in both of the tumour-bearing groups (W and LW), a leucine-rich diet maintained protein synthesis and reduced protein degradation in the LW group. This observation suggests that the LW group had a less intense catabolism of lean body mass, as was verified in the W group, which was likely related to the degradation of foetal muscle protein that was enhanced by oxidative stress [38,45]. According to our previous studies, lean body mass was reduced during tumour growth, and dietary supplementation with leucine minimised this loss [3,5,8,24]. Intense protein catabolism, primarily in the skeletal muscle, may have provided substrates, predominantly amino acids and gluconeogenic substrates, to be used by neoplastic cells [25,46,47]. Consequently, it is thought that tumour development induces a deviation of nutrients from the placenta and foetus (which is the reason for the decrease in foetal weight and reduction in the foetus/placenta ratio that is associated with increased protein degradation and oxidative stress). In parallel, the foetuses from mothers that were subjected to graduated food restriction (Cp and Lp) showed reduced weight, but their serum parameters and total muscle protein were similar to those of the control group animals. Nutritional supplements are particularly important for foetal viability and foetus/placenta unity, but foetuses have the capacity to adapt metabolically to acute and chronic changes in maternal and foetal serum nutrients content [48,49]. In contrast, we show that foetuses from tumour-bearing mothers, primarily those in group W, were not able to efficiently adapt their metabolism under cancer conditions compared to those under food restriction, as found in pair-fed foetuses (Cp). This fact may be related to the action of substances that are produced by cancer cells, as we have previously reported [4,7,50,51]. Carbo and colleagues [52] found deficits in the amino acid mobilisation of foetuses in pregnant rats that received tumour necrosis factor during pregnancy, and this was associated with decreased placental blood flow due to the action of this cytokine. We previously verified that pregnant rats that were injected with ascitic liquid (obtained from tumour-bearing animals without tumour cells) exhibited an increase in foetal reabsorption and a reduction in foetal and placental weight; similar deleterious effects were found in tumour-bearing mothers [3,4,50]. In these experiments, we proposed that these changes were induced by substances produced by cancer cells and did not result from only the effect of reducing nutrients, as was observed in this study when comparing the W and Cp foetuses.

Pregnancy changes, especially the increase in muscle protein breakdown, are related to the effects of the tumour on the body. In the cancer host, the catabolism that is associated with the reduction and/or maintenance of the protein synthesis process is increased [24,53] and is related to increased oxidative stress [38,45,50]. In this case, the foetuses from the rats in group W displayed reduced protein synthesis, which was also associated with an increase in proteolysis caused by the activation of proteolytic systems and a high oxidative stress process. The ubiquitin-proteasome pathway can be modulated by a leucine-rich diet [6,8]. As has been reported in many other studies, the activity of this process was higher and more widely observed in foetal cells. The 20S subunit, which comprises the catalytic core of the ubiquitin-proteasome proteolytic pathway, is most likely involved in protein turnover, leading to differentiation and development of embryonic and foetal tissues. However, we showed that although the 20S subunit was expressed at lower levels in the W and LW foetuses, the greater chymotrypsin-like activity and oxidative stress that were found in group W were sufficient to promote protein wasting in those foetuses. More importantly, under leucine nutritional supplementation, the LW foetuses showed less increased protein degradation because leucine diminished the increase in chymotrypsin activity and maintained the cathepsin enzyme while increasing the antioxidant response; these results suggest a beneficial effect of leucine in minimising proteolysis. Moreover, tumour growth produces alterations that affect placental metabolism and interact with the placenta-foetus, as evidenced by intrauterine growth restriction with increased foetal-muscle catabolism [3,4,6,24,52].

Nutritional supplementation with leucine provides several benefits by activating cellular synthesis processes [6,24,25]. These data suggest that the foetuses that received nutritional leucine supplementation, even those that were suffering from their mother’s tumours, showed diminished deleterious effects due to tumour growth compared to those that did not receive leucine; the foetuses in the LW group had similar muscle protein synthesis to the control foetuses, and more importantly, the process of protein catabolism was minimised and was equal to that of the control group [4,5,54]. Further studies are underway in our laboratory to determine whether leucine supplementation can counteract the damage to foetal growth during cancer development and the mechanism underlying this effect. These data indicate the importance of co-adjuvant therapy in improving the state of host responses to clinical treatment and particularly the importance of leucine supplementation, which has a positive effect on the host mother and on foetal development.

## Conclusions

The nutritional supplementation of a leucine-rich diet induced protein synthesis in the skeletal muscle of foetuses of pregnant, tumour-bearing rats. This effect may be due to minimisation of protein catabolism and enhancement of the anti-oxidative response.

## Competing interests

The authors declare that there is no competing interest that could be perceived as prejudicing the impartiality of the research reported.

## Authors’ contributions

BLGC contributed to the data collection, analysis, data interpretation and manuscript preparation. MCCGM conceived the study and its design and coordinated the work and the preparation of the manuscript. Both authors read and approved the final manuscript.
